# Time-resolved *in situ* vibrational spectroscopy for electrocatalysis: challenge and opportunity

**DOI:** 10.3389/fchem.2023.1231886

**Published:** 2023-07-27

**Authors:** Danya Lyu, Jinchang Xu, Zhenyou Wang

**Affiliations:** ^1^ GBA Branch of Aerospace Information Research Institute, Chinese Academy of Science, Guangzhou, China; ^2^ Guangdong Provincial Key Laboratory of Terahertz Quantum Electromagnetics, Guangzhou, China

**Keywords:** time-resolved spectroscopy, *in situ*, infrared, Raman, ATR-SEIRAS, SERS

## Abstract

Understanding the structure-activity relationship of catalysts and the reaction pathway is crucial for designing efficient, selective, and stable electrocatalytic systems. *In situ* vibrational spectroscopy provides a unique tool for decoding molecular-level factors involved in electrocatalytic reactions. Typically, spectra are recorded when the system reaches steady states under set potentials, known as steady-state measurements, providing static pictures of electrode properties at specific potentials. However, transient information that is crucial for understanding the dynamic of electrocatalytic reactions remains elusive. Thus, time-resolved *in situ* vibrational spectroscopies are developed. This mini review summarizes time-resolved *in situ* infrared and Raman techniques and discusses their application in electrocatalytic research. With different time resolutions, these time-resolved techniques can capture unique dynamic processes of electrocatalytic reactions, short-lived intermediates, and the surface structure revolution that would be missed in steady-state measurements alone. Therefore, they are essential for understanding complex reaction mechanisms and can help unravel important molecular-level information hidden in steady states. Additionally, improving spectral time resolution, exploring low/ultralow frequency detection, and developing operando time-resolved devices are proposed as areas for advancing time-resolved techniques and their further applications in electrocatalytic research.

## 1 Introduction

Electron-transfer reactions at the electrode surface can be categorized as inner- or outer-sphere reactions. For an outer-sphere reaction, the electrons transfer tunnelling through a solvent layer, thus, no direct chemical interaction between the electrode and active species occurs. Therefore, the electron-transfer rate is exponentially increased with the applied overpotential. In contrast, electrocatalysis involves typical inner-sphere reactions where the electron-transfer rate is highly rated to the surface structure of the catalyst and reaction pathway, in addition to the applied overpotential making it far more complicated than an outer-sphere reaction.

The rational design of highly efficient electrocatalytic systems requires a profound awareness of the structure-activity relationship of the catalysts and the reaction pathway. Theoretical simulations such as density functional theory (DFT) can provide some cues of the critical adsorption sites and interface structures. Morphology and structure detection techniques such as scanning electron microscopy, transmission electron microscopy, atomic force microscopy, X-ray absorption, X-ray diffraction, X-ray photoelectron spectroscopy, electron paramagnetic resonance, Mössbauer spectroscopy, etc., have been applied both *in situ* and *ex situ* to monitor the structure evolution, coordination environment, valence state, electronic property, etc., (Zhang et al., 2019; Chen et al., 2023). However, direct spectral evidence of molecular-level factors is still challenging to obtain. Therefore, *in situ* vibrational spectroscopy including Raman, Infrared (IR), and sum frequency generation (SFG) spectroscopy able to capture the molecular fingerprint information are necessary (Xu et al., 2021; Liu et al., 2022). SFG, a non-linear spectroscopy, benefits from the specific interfacial selection rules and is easy for pump-probe time-resolved studies. Recently, phase-sensitive second-harmonic generation, a specific sum frequency nonlinear effect, was developed to measure the electrochemical potential of zero charge at the Pt-water interface (Xu et al., 2023). But these techniques highly rely on well-trained specialists and sophisticated devices thus, hasn’t been widely applied yet (Li et al., 2022). This mini review focuses on the mostly used time-resolved *in situ* strategies of IR and Raman spectroscopies and discusses their applications in electrocatalytic research.

Herein, “*in situ*” refers to the measurements performed during the reaction under relevant reaction conditions. In comparison, “operando” measurements are taken under reaction conditions similar to those of realistic reactors. (Bañares, 2005; Yang et al., 2021). As a powerful tool, the *in situ* vibrational spectra are typically obtained under a series of preset potentials with stable currents as a steady-state method. Highly reproducible spectra and rich information such as the structure of the catalysts, adsorbed intermediates, structure of the interfacial solvents, et al. are obtained (Zhu et al., 2020; He et al., 2023). However, valuable transient information such as the dynamic kinetic, relaxation of the interfacial structure, and ultrashort lived intermediate is inevitably missed by using the steady-state measurement alone. Hence, various time-resolved IR and Raman spectroscopic techniques with different time resolutions are developed.

## 2 Time-resolved *in situ* fourier-transformed infrared (FTIR) study

Infrared spectroscopy exploits the specific absorption of infrared radiation at characteristic frequencies of molecular vibrations. It has been widely used to monitor functional groups, molecular symmetry, and interactions between catalysts and molecules during electrochemical reactions (Chalmers and Griffiths, 2002). External reflection and internal reflection also known as attenuated total reflection (ATR) are two main types of detection modes used in *situ* measurements. As surface-enhanced infrared absorption has been proved on many important transient metals such as Cu and Pt, that surface-enhanced infrared reflection absorption spectroscopy (SEIRAS) coupled with ATR detection mode is widely used in spectroelectrochemistry (Liu et al., 2021; Cuesta, 2022).

**FIGURE 1:**
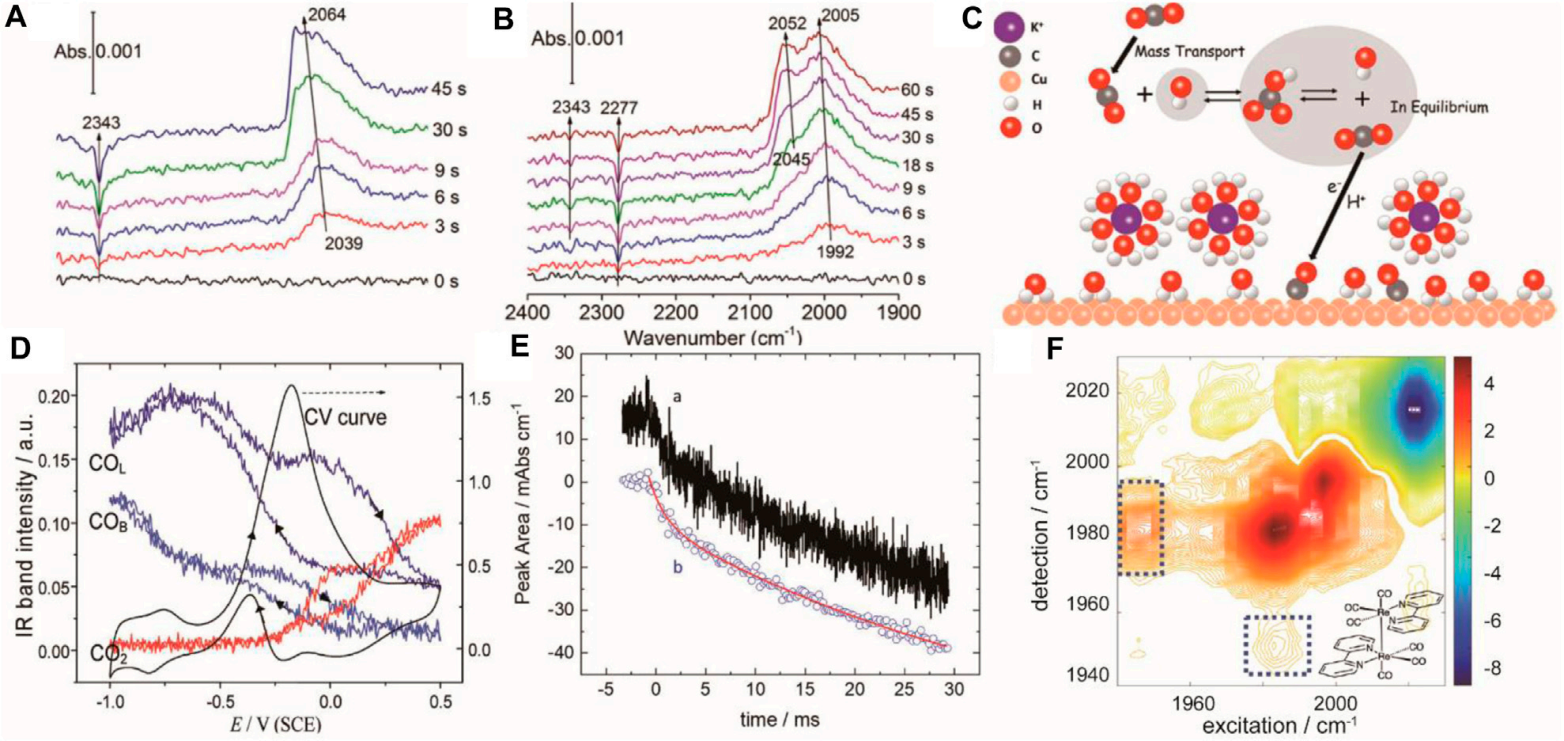
Real-time ATR-SEIRAS spectra recorded after stepping the Cu thin film electrode to −0.6 V in a CO_2_ saturated 0.1 M KH^12^CO_3_ **(A)** and KH^13^CO_3_ **(B)** solution. Adapted with permission from (Zhu et al., 2017). Copyright 2017 American Chemical Society; **(C)** schematic diagram of equilibrium between CO_2_ and bicarbonate anions in the electrolyte during the CO_2_RR. Adapted with permission from (Zhu et al., 2017). Copyright 2017 American Chemical Society. **(D)** IR intensity of CO_L_, CO_B_, and CO_2_ with the CV. Adapted with permission from (Li et al., 2012). Copyright 2012 American Chemical Society **(E)** Dual comb IR spectra of integrated peak area of 4-dimethylaminopyridine at 1,628 cm^−1^ with 20 μs **(A)** and 200 μs **(B)** time binning. Adapted with permission from (Lins et al., 2020). Copyright 2020 American Chemical Society; **(F)** Difference 2D-IR spectra of Re (bpy) (CO)_3_Cl at −1.6 V. Adapted with permission from (Kiefer et al., 2021). Copyright 2021 American Chemical Society.

Linear scan mode is provided by commercial FTIRs. As it is easy to implement without any additional accessory, it is widely used to monitor electrochemical reactions under a second-time regime. As an example, Zhu et al. studied the CO_2_RR on the Cu thin film combining real-time ATR-SEIRAS with isotopic labeling (Zhu et al., 2017). Surface ^12^CO_2_ and surface adsorbed ^12^CO were observed in the KH^12^CO_3_ electrolyte saturated with ^12^CO_2_ (Figure 1A). Interestingly, new peaks assigned to surface ^13^CO_2_ and adsorbed ^13^CO were observed in the KH^13^CO_3_ electrolyte saturated with ^12^CO_2_ (Figure 1B) indicating the existence of an equilibrium between CO_2_ and bicarbonate anions in the electrolyte. This result provided that the CO_2_ source of CO_2_RR is from the surface equilibrium rather than the free CO_2_ (Figure 1C). The drawback of linear scan is that it is unable to catch up with fast electrochemical reactions as each spectrum takes hundreds of ms to collect (Pérez-Martínez et al., 2021). Combing *in situ* ATR-FTIR with on-line mass spectrometry can capture the reaction kinetics of surface species and volatile products simultaneously, helping to evaluate the contribution of partial reactions (Heinen et al., 2007a; Heinen et al., 2007b ). Besides, polarization modulation is applied in time-resolved infrared reflection absorption measurement to character the coordination and symmetry of surface species such as cyanide (Hosseini et al., 2018 ).

High temporal-resolution detection methods with ms and μs resolution based on the FT-IR had been developed. One is rapid-scan time-resolved FT-IR which employs moving mirrors to reduce the scan time to the ms scale and is useful for monitoring processes with a half-life time 100 ms (Li et al., 2012). An example of this technique’s application is the oxidation of methanol on a Pt microelectrode in a thin-layer cell combined with external reflection detection mode (Zhou et al., 2004). To overcome the low mass transport rate, a Pt microelectrode and a flow cell were specially designed to reduce the electrode time constant down to 100 μs. Cyclic voltammograms and IR spectra can be recorded simultaneously at a high potential scan rate up to 200 mV/s (Figure 1D). The study observed linearly bonded CO (CO_L_) and bridge-bonded CO (CO_B_) under −0.17 V and −0.54 V vs. SCE respectively. Furthermore, the difference in potential between CO_L_ and CO_B_ indicates that the activation energy of methanol oxidation via CO_B_ is lower than CO_L_, revealing a dual reaction mechanism.

Another approach is the step-scan method where the moving mirror is moved in a series of fixed steps. At each step, a full interferogram is collected and Fourier transformed into a single spectrum point. The time delay between successive interferograms is controlled by the rate of movement of the moving mirror. The resulting two-dimensional data arrays consisting of time-delayed interferograms at each wavenumber can be used to generate kinetic information. (Ataka et al., 1999). Osawa et al. first reported the *in situ* step-scan time-resolved FTIR with a sub-millisecond resolution to monitor a one-electron reduction of heptylviologen (Osawa et al., 1994). Zhou et al. reported the monitoring of CO oxidation on Pt microelectrode, an irreversible reaction by using a thin layer cell at a time resolution of 250 μs (Zhou et al., 2005).

Instead of using the moving mirror in the step-scan method, as an alternative, dual frequency comb IR laser spectroscopy achieved microsecond time resolution by using a heterodyned detector to record the interference signal generated by two IR laser combs (Lins et al., 2020). Eliminating the need for successive mirror movements greatly reduces the sampling time by two orders of magnitude. In a recent study, transient evolution of 4-dimethylaminopyridine on the electrode surface was investigated through ATR-SEIRAS mode. Time-resolved integrated peak area of 1,628 cm^−1^ was recorded and fitted by a double exponential function of time to calculate the surface diffusion coefficient (Figure 1E). This method achieved the time resolution of 10 μs and a detection limit of 5% of a monolayer. Additionally, another ultrafast technique, time-resolved 2D-IR, can display both frequency and time domain information. It involves the excitation of a sample with two laser pulses separated by a time delay, followed by detection of the emitted IR radiation at different frequencies and time delays. It has been used for *in situ* monitoring of CO_2_RR using Re (bpy) (CO)_3_Cl as the catalyst based on the transmission mode. The reaction intermediate, Re-Re dimer, was directly observed from the difference spectrum (Figure 1F).

## 3 Time-resolved *in situ* Raman study

As a complementary of IR, Raman spectroscopy is also used to identify functional groups in a molecule involving changes in polarizability (Chalmers and Griffiths, 2002). The weak Raman signal of water makes Raman spectroscopy a powerful tool to monitor aqueous reactions. Surface-enhanced Raman spectroscopy (SERS), a near-field effect, meriting from the electromagnetic field and chemical enhancement, is suitable to detect surface information such as interfacial composition and adsorbents. Usually, the signal of normal Raman is weak so SERS or resonance Raman is frequently used in time-resolved studies (McCreery and Packard, 1989).

An early approach to achieve microsecond time resolution in electrochemical measurements was reported by using a potential averaging method equipped with a charge-coupled device (CCD) (Tian et al., 1991; Tian et al., 1996). In this method, a square-wave potential modulation was applied to the electrode, and the obtained spectrum contains all the average information at two potentials. The signal of each potential was separated through deconvolution. This technique offers improved time resolution compared to the response time of CCD as the time resolution solely depends on the electrochemical response. Important surface structures such as the potential related adsorption orientation and SERS active sites can be obtained.

**FIGURE 2:**
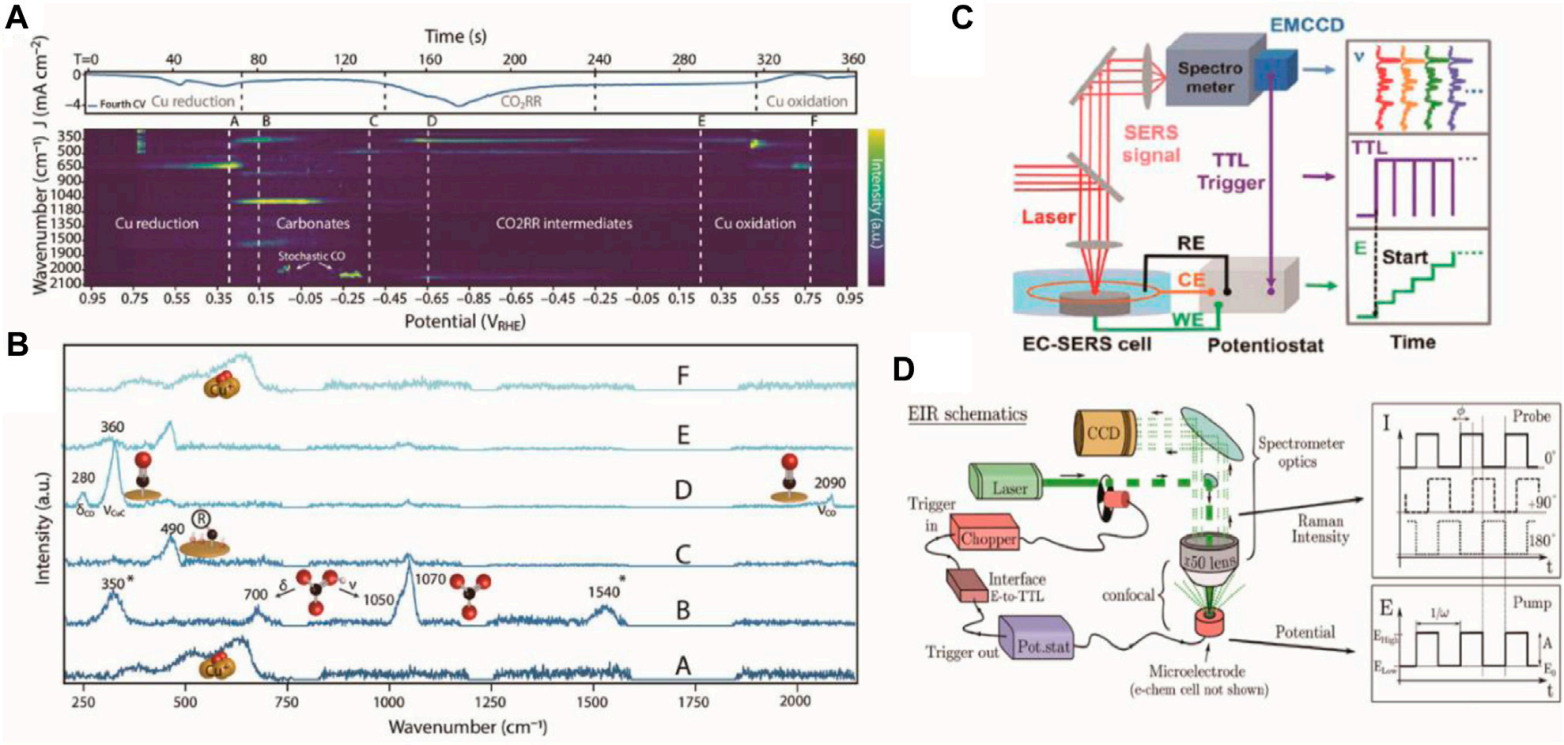
**(A)** Time-resolved SERS data of electrodeposited copper during the cyclic voltammetry at 10 mV/s. Adapted with permission from (de Ruiter et al., 2022). **(B)** Raman spectra of typical moments corresponding to the dashed lines in **(A)**. Adapted with permission from (de Ruiter et al., 2022). **(C)** Scheme of transient electrochemical SERS and the synchronization sequence of the trigger. (Zong et al., 2015). Copyright 2015 American Chemical Society. **(D)** Scheme of the pump-probe mode setup with the modulation of Raman intensity and potential vs. time. Adapted with permission from (D'Amario et al., 2022).

An alternative method for obtaining time-resolved spectra in electrochemical measurements is to perform simultaneous sampling of Raman and electric signals during a transient electrochemical test. Gao et al. first utilized this approach to demonstrate time-resolved SERS in conjunction with a cyclic voltammetry test using a spectrograph-multichannel detector. The potential sweep rate was 5 mV/s and spectral integration time was 5 s due to the limited sensitivity of the detector at the 1980 s (Gao et al., 1988). Ruiter et al. adopted this approach to investigate the CO_2_ reduction reaction on Cu (oxide) electrode during the cyclic voltammetry. They used a potential sweep rate of 10 mV/s and spectral sampling time of 1 s to obtain vibration modes of copper oxides, carbonate/bicarbonate, and CO. (Figures 2A, B) (de Ruiter et al., 2022). Furthermore, they observed evidence of low-overpotential CO_2_-to-CO activation. Despite applying a low potential scan rate, the Raman signal remained a potential-average result as the spectra sampling rate was longer than the potential scan. With the rapid development of photoelectric detector, millisecond-resolved SERS was illustrated by Zong et al., equipped with a high-speed readout EMCCD that was trigged synchronically by the potentiostat without repetitive cycled acquisitions (Figure 2C) (Zong et al., 2015). In this manner, the Raman signal and current were precisely correlated. Most recently, stimulated Raman spectroscopy, a three-order nonlinear effect, was deployed to monitor species near the electrode’s surface during a redox model reaction with a millisecond-to-second resolution, helping to overcome the limitation that only a few electrode materials are SERS active (Xu and Suntivich, 2023).

Pump-probe method, a general technique applied in ultrafast time-resolved spectroscopy, is also applied in electrochemical research. However, instead of using pump light, electrochemical tests like chronoamperometry and potential sweep typically serve as the trigger for reactions. Due to the limited sampling time of a typical CCD (∼100 ms), gated detectors are generally employed for high time-resolved experiments. (Shi et al., 1990, 1997). Previous studies have reported sub-millisecond resolution systems equipped with a nanosecond pulse laser and an ICCD camera, wherein the formation of monocation radical of heptylviologn was monitored (Misono et al., 1993). More recently, D'Amario et al. provided a simple modification of a commercial confocal Raman system with a time resolution of sub-microsecond (0.6 ms) by a CCD camera. A high-frequency square wave step was used to trigger the reaction, while a modulated continuous laser served as the detection light. (Figure 2D) (D'Amario et al., 2022). By isolating relevant difference bands of transient species from those throughout whole pumping frequencies, they could obtain a detailed understanding of the reaction mechanism. Nevertheless, the pump-probe method’s drawback is that the system must at least partially revert to its initial state.

## 4 Outlook

In this mini review, we provide a summary of the current instrumental methods of time-resolved vibrational spectroscopies and their applications in electrocatalysis research. With the rapid development of photoelectric detectors, there are immense opportunities to improve our understanding of electrocatalytic energy conversion. Herein, three main opportunities and challenges are addressed.

(1) Improve spectral time resolution.

To investigate short-lived intermediates in electrocatalytic reactions, it is necessary to employ time-resolved techniques with a temporal resolution of milliseconds to microseconds. Distinguishing between active and poisoned intermediates is important for understanding the pathway of electrocatalytic reaction. Time-resolved techniques can monitor intermediate generation, decay, and conversion processes, enabling determination of reaction kinetics and intermediate lifetimes, thus, revealing reaction kinetics. Microsecond time-resolved vibrational spectroscopy techniques still remain challenging. At this time scale, electrochemical process of the system needs to be carefully cheeked. For example, the charging process of the electric double layer controls the RC time constant that determines the test time zone. The mass transport process affects the thickness of the diffusion layer and kinetic behaviour according to Fick’s law. Thus, flow cells and ultramicroelectrodes are particularly designed and used to reduce the RC time constant. Besides, charge transfer is another important process in electrocatalytic reactions. To probe such ultrafast processes under the ns-fs scale, ultrafast techniques are required such as the pump-probe, and pump-push-probe strategies that pulse laser acts as the trigger instead of the potential. The limitation lies in the potentiostat, as the response time of commercial potentiostat is much longer than the laser that customized potentiostat is needed (Zwaschka et al., 2021).

(2) Explore low/ultralow frequency detection.

The low and ultralow frequency range is crucial to understanding the structural and interactional information of catalysis, such as adsorption bonds, surface lattice species, and local strain, but has been seldom explored. Low frequency detection is quite difficult for ATR-SEIRAS due to the strong optical absorption of the conventional Si prism used in the electrolytic cell. Recently, a micromachined Si wafer window was developed that extends the detection window of ATR-SEIRAS down to 650 cm^−1^ which was applied to probe the surface structure and additives (Ma et al., 2020; Mao et al., 2022). However, the detection below 650 cm^−1^ is even quite challenging due to the lack of appropriate IR sources. Recently, synchrotron radiation and free electron laser with tenable wavelength and ps resolution has been launched which makes a great opportunity for low frequency detection and time-resolved research (Ye et al., 2016). Both synchrotron radiation source and free electron laser are served as national projects, not easy to access for a common lab. On the contrary, the ultralow frequency (5 ∼ 200 cm^−1^) detection of Raman is much easier to implement as there are commercial BragGate notch filters to choose from. Taking advantage of the ultralow-frequency Raman, direct observation of structural changes (photon mode) of the metal clusters became possible (Kato et al., 2020). It can also be used to observe the extra molecular vibration mode of surface adsorption species revealing the specific structural and environmental information of electrode-electrolyte interface (Inagaki et al., 2017; Kondo et al., 2022).

(3) Develop new methods and devices under operando conditions.

During the *in situ* experiment, the mostly used spectral electrolytic cell and electrode such as a thin layer electrolytic cell and metal electrode, are typically designed for lab-scale research, which is far from industrial usage. Operando experiment is required to bridge the gap between lab research and realistic scenarios. One approach is to design special electrolytic cells that meet the optical detection requirements and also provide similar working conditions as realistic devices. For example, systematic operando studies of membrane electrodes are still lacking to correlate liquid-gas-solid multiphase information (Li et al., 2022). Another approach is to design operando optical sensors (Huang et al., 2022), such as a hollow-core optical fiber-based Raman probe can be embedded into a Li-ion pouch cell for the operando detection of liquid electrolyte species (Miele et al., 2022) and chalcogenide glass fiber-based IR probes can directly traverse through commercial Na (Li)-ion batteries for the real-time monitoring of electrolyte evolution (Gervillié-Mouravieff et al., 2022). These pioneer works provide inspired insights and opportunities for the development of vibrational spectroscopy tools for online simultaneous investigation under real-operational conditions.
